# N-Terminal Amino Acid Affects the Translation Efficiency at Lower Temperatures in a Reconstituted Protein Synthesis System

**DOI:** 10.3390/ijms25105264

**Published:** 2024-05-12

**Authors:** Tomoe Fuse-Murakami, Rena Matsumoto, Takashi Kanamori

**Affiliations:** GeneFrontier Corporation, 273-1 Kashiwa, Kashiwa-shi 277-0005, Chiba, Japan

**Keywords:** cell-free protein synthesis, PURE system, N-terminal sequence, translation efficiency, early elongation

## Abstract

The *Escherichia coli* (*E. coli*)-based protein synthesis using recombinant elements (PURE) system is a cell-free protein synthesis system reconstituted from purified factors essential for *E. coli* translation. The PURE system is widely used for basic and synthetic biology applications. One of the major challenges associated with the PURE system is that the protein yield of the system varies depending on the protein. Studies have reported that the efficiency of translation is significantly affected by nucleotide and amino acid sequences, especially in the N-terminal region. Here, we investigated the inherent effect of various N-terminal sequences on protein synthesis using the PURE system. We found that a single amino acid substitution in the N-terminal region significantly altered translation efficiency in the PURE system, especially at low temperatures. This result gives us useful suggestions for the expression of the protein of interest in vitro and in vivo.

## 1. Introduction

The protein synthesis using recombinant elements (PURE) system is a reconstituted cell-free protein synthesis system based on purified factors necessary for *Escherichia coli* (*E. coli*) translation. The major advantage of the system is that it consists only of purified factors that are required for translation, and it is devoid of nucleases, proteases, and other unrelated factors, such as metabolic enzymes [1]. The PURE system is currently used not only for studying the mechanism of translation and protein folding [2,3,4,5,6,7,8], but also in synthetic biology and protein engineering applications [9,10,11,12,13,14,15,16]. The yield of the proteins synthesized using the PURE system was initially lower than that of the *E. coli* S30 extract system, but it has now increased to 1 mg/mL following optimization of the reaction composition [17]. However, the yields are highly dependent on the protein being synthesized, and, therefore, it is necessary to identify sequences that would increase protein synthesis using the PURE system.

The efficiency of translation on the ribosome is affected by the nucleotide and amino acid sequences. In particular, the nucleotide and amino acid sequence in the N-terminal region significantly affects translational efficiency [18,19,20,21,22,23,24]. Most of the studies focused on the identification of the optimal sequence are based on protein expression experiments performed in *E. coli* cultured at 37 °C. In the case of the expression in *E. coli*, the host proteins are also expressed. In contrast, in the PURE system, only the protein of interest is synthesized, and therefore the optimal sequence may be different based on the inherent effect of such a sequence. Also, protein synthesis using the PURE system is generally performed at 37 °C. However, protein synthesis at 37 °C using the PURE system often leads to aggregation of the synthesized proteins [5]. In such cases, the reactions are occasionally performed at 30 °C or lower temperatures to prevent aggregation of the synthesized proteins. Therefore, the sequence needs to be optimized to increase the efficacy of protein synthesis at temperatures lower than 37 °C.

In this study, we investigated the effect of the N-terminal sequence on protein synthesis in the PURE system. We found that a single amino acid substitution in the N-terminal region significantly altered the translation efficiency in the PURE system, especially at low temperatures, which gives us useful suggestions for the expression of the protein of interest in vitro and in vivo.

## 2. Results

### 2.1. Synthesis of Model Proteins at Different Temperatures

**Figure 1:**
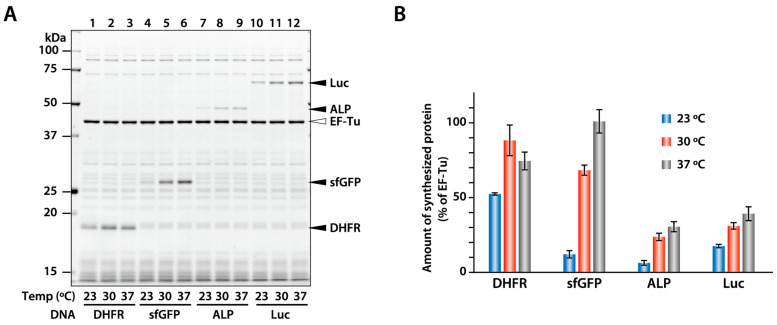
Synthesis of four model proteins at 23, 30, and 37 °C. (**A**) DHFR, sfGFP, ALP, and Luc were synthesized using the PURE system at 23, 30, and 37 °C for 24 h. The reaction mixture was separated through SDS-PAGE. The synthesized products and internal elongation factor Tu (EF-Tu) are indicated with arrowheads. (**B**) The intensity of the product bands was quantified, and the amount of product was calculated as the ratio of EF-Tu in the reaction mixture. The average of three independent experiments is shown. Error bars indicate standard deviation (S.D).

Firstly, we synthesized four model proteins, including dihydrofolate reductase (DHFR) and alkaline phosphatase (ALP) from *E. coli* and superfolder green fluorescent protein (sfGFP) and firefly luciferase (Luc), using the PURE system at 23, 30, and 37 °C to determine the effect of synthesis temperature on synthesis efficacy (Figure 1). To reduce the influence of codon usage, the DNA sequences of Luc and sfGFP, which were derived from organisms other than *E. coli*, were designed based on the codon usage frequency in *E. coli* [25]. When the four proteins were synthesized at 37 °C, the yields of DHFR and sfGFP were markedly greater than those of Luc and ALP. However, the amount of sfGFP synthesized at 23 °C was lower than that of DHFR and Luc. The amounts of sfGFP and ALP synthesized at 23 °C were reduced to 12% and 20% compared with those at 37 °C, respectively. In contrast, the amounts of DHFR and Luc synthesized at 23 °C were 70% and 45% compared with those at 37 °C, respectively. These results indicate that the decrease in the synthesis efficiency at lower temperatures does not correlate with the productivity at 37 °C.

### 2.2. Synthesis of sfGFP with a Different Fourth Amino Acid

**Figure 2:**
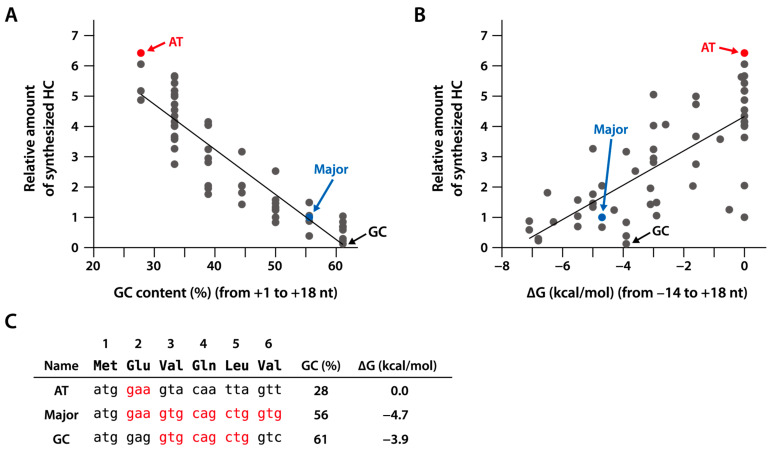
Synthesis of trastuzumab-heavy chain with different N-terminal nucleotide sequences. Fifty-eight constructs of trastuzumab-heavy chain (HC; VH + CH1) with a different N-terminal nucleotide sequence were synthesized at 30 °C for 4 h using the PURE system in the presence of 3 mM of oxidized glutathione, 4 µM of DsbC, 5 µM of DnaK, 1 µM of DnaJ, and 1 µM of GrpE. The reaction mixture (0.25 µL) was subjected to SDS-PAGE, and the gel was stained with Oriole (Bio-Rad, Hercules, CA, USA). The band intensity of the synthesized HC variants was measured using an ImageQuant LAS imager (Cytiva, Tokyo, Japan), and the relative amount was calculated as a ratio of the amount of major variants (Major). (**A**) The relative amounts of synthesized HC variants were plotted against the GC content of the nucleotide sequence encoding the first six amino acids. (**B**) The relative amount of the synthesized HC variants was plotted against the minimum free energy (MFE, ∆G) of the nucleotide sequence around the start codon. ∆G was calculated using the RNAfold web site (http://rna.tbi.univie.ac.at/cgi-bin/RNAWebSuite/RNAfold.cgi (accessed in November 2017)). (**C**) Nucleotide sequences of the AT variant (AT; with the highest yield), GC variant (GC; with the lowest yield), and major variant (Major) with only the most frequent *E. coli* codons. The most frequently used codons in *E. coli* are shown in red letters.

Translation efficiency is affected by the amino acid and nucleotide sequences in the N-terminal region of the open reading frame (ORF) rather than by those along the entire ORF [18,19,20,21,22,23,24]. Nucleotide sequences with AT-rich codons in the N-terminal region are translated preferentially [18]. These sequences destabilize the secondary structure of mRNA around the initiation codon. We verified whether the same results hold true in the PURE system. Fifty-eight variants with six-codon substitutions in the N-terminal region of the trastuzumab-heavy chain [26] were synthesized using the PURE system, and their yields were compared (Figure 2). The AT-rich codon variant resulted in the largest protein amount and differed (by >50-fold) from the GC-rich codon variant, which yielded the lowest amount. This indicates that AT-rich codons in the N-terminal region are preferred for protein synthesis in the PURE system.

**Figure 3:**
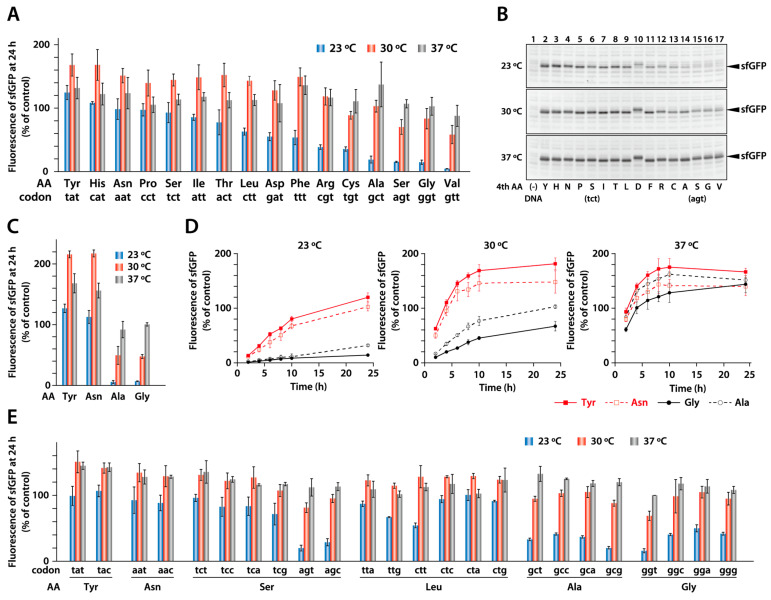
Synthesis of sfGFP variants with a different amino acid at the fourth position. (**A**) sfGFP was synthesized at 23 °C (blue bar), 30 °C (red bar), and 37 °C (gray bar) for 24 h with the indicated amino acid at the fourth position. The fluorescence intensity of the synthesized products was measured. (**B**) The synthesized products were analyzed using SDS-PAGE. The band corresponding to sfGFP is indicated with an arrowhead. (**C**) sfGFP containing tyrosine, asparagine, alanine, or glycine at the fourth position was synthesized from mRNA at 23 °C (blue bar), 30 °C (red bar), and 37 °C (gray bar) for 24 h. The fluorescence of the synthesized products was measured. (**D**) Time course of the synthesis reaction. sfGFP with glycine (black line), alanine (black dotted line), tyrosine (red line), or asparagine (red dotted line) was synthesized at 23, 30, and 37 °C, and the fluorescence intensity of the synthesized products was measured at 2, 4, 6, 8, 10, and 24 h. (**E**) sfGFP containing a synonymous codon for tyrosine, asparagine, serine, leucine, alanine, or glycine at the fourth position was synthesized at 23, 30, and 37 °C for 24 h, and the fluorescence of the product was measured. In all experiments, the relative intensity was calculated as a ratio of the fluorescence of wild-type sfGFP (glycine (ggt codon) at the fourth position) at 37 °C. The average of three independent reactions is shown. Error bars indicate standard deviation (S.D).

In the above experiment, we used sfGFP DNA with AT-rich codons in its N-terminal region. Therefore, next, we focused on the amino acid sequence. Immediately following the first methionine, amino acids with aromatic rings or basic side chains, such as phenylalanine and lysine, tend to increase the translational efficiency [20,27]. In the N-terminal region of sfGFP, glycine, the smallest amino acid, is located at the fourth position. To examine the effect of this residue, we synthesized mutant versions of sfGFP in which the fourth glycine residue was replaced with other amino acids. To retain a high AT content, we used template DNAs in which the GGT codon for the fourth glycine was replaced by other NNT codons. We synthesized 16 sfGFP variants, including a wild-type version with glycine, using the PURE system at 23, 30, and 37 °C and then measured the fluorescence of the products (Figure 3A). Analysis of the protein products using SDS-PAGE also confirmed that the fluorescence intensity correlated with the amount of the synthesized product (Figure 3B). The results showed that the amount of product varied depending on the amino acid at the fourth position, particularly at lower temperatures.

When the sfGFP variants were synthesized at 37 °C, the fluorescence intensity differed by approximately 49% between the variants (Figure 3A, gray bar). However, when the synthesis was performed at 30 or 23 °C, the fluorescence intensity differed significantly (up to 3- or 28-fold, respectively) between the different variants, with tyrosine showing the highest yield and valine showing the lowest yield (Figure 3A, red and blue bar). Substitution with a larger amino acid, such as tyrosine, histidine, or asparagine, at the fourth position led to higher synthesis efficiency at 23 °C compared to that with smaller amino acids, such as glycine, valine, and alanine. Interestingly, the variants with high synthesis efficiency at 23 °C also exhibited higher synthesis efficiency at 30 °C than at 37 °C, as in the case of DHFR (shown in Figure 1). The synthesis reaction performed from mRNA yielded results similar to those obtained with DNA (Figure 3C). This indicates that the difference in the protein yield of the variants is primarily because of the differences in translation and not transcription.

We measured the time course of the synthesis reaction using four variants (Figure 3D). The tyrosine and asparagine variants, which showed high productivity after 24 h, also exhibited faster initial rates. This result suggests that the rate of peptide bond formation determines the final productivity of the sfGFP variants.

### 2.3. Effect of a Synonymous Codon at the Fourth Position

As mentioned earlier, translation efficiency depends on AT content in the N-terminal region. Therefore, we compared the productivity of several variants that were substituted with synonymous codons other than NNT (Figure 3E). For the tyrosine and asparagine variants with synonymous codons, the difference in fluorescence intensity was negligible. In contrast, for the glycine and leucine variants, the synthesis efficiency at 23 °C differed depending on the codon. Serine is encoded by six codons, including four TCN and two AGN codons. Interestingly, we found that the TCN codon yielded a higher amount of product at 23 °C, whereas the AGN codon resulted in a lower yield. These results indicate that the effect of codon selection depends on the amino acid, but the difference in amino acids at the fourth position has a more significant effect on sfGFP productivity.

### 2.4. Synthesis of sfGFP with Different Amino Acids at the Second Position

**Figure 4:**
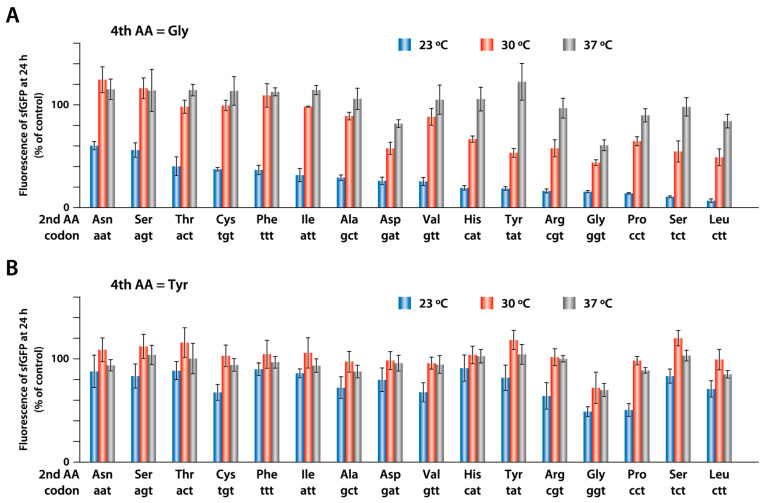
Synthesis of sfGFP containing a different amino acid at the second position. sfGFP with the indicated amino acid at the second position along with glycine (**A**) or tyrosine (**B**) at the fourth position was synthesized at 23, 30, and 37 °C for 24 h. The fluorescence of the synthesized products was measured, and the relative intensity was calculated as a ratio of the fluorescence of sfGFP containing serine (TCT codon) at the second position synthesized at 37 °C. The average of three independent reactions is shown. Error bars indicate standard deviation (S.D).

Several studies have reported that the second amino acid following the initiator methionine affects the translation efficiency [18,23,24]. We compared the synthesis efficiencies of sfGFP variants with different amino acids at the second position. Substitutions at the second amino acid were based on two sfGFP variants with different amino acids at the fourth position: one with tyrosine (high productivity), and the other with glycine (low productivity). When the fourth amino acid was glycine, translation efficiency varied depending on the second amino acid (Figure 4A). The productivity at 23 °C differed nine-fold between the asparagine and leucine variants. In contrast, when the fourth amino acid was tyrosine, the differences in the synthesis efficiency of the different second amino acids were small (Figure 4B). These results suggest that in the case of sfGFP, the effect of the fourth amino acid on productivity at low temperatures was greater than that of the second amino acid.

### 2.5. Effect of Addition of N-Terminal Sequence on the Productivity of Other Proteins

**Figure 5:**
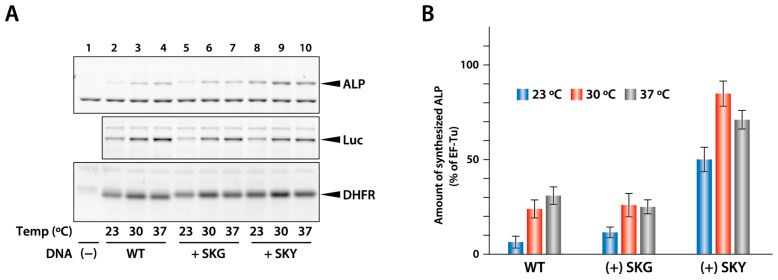
Synthesis of model proteins with the addition of the N-terminal sequence of sfGFP. (**A**) DHFR, Luc, and ALP, in which the Ser-Lys-Gly (SKG) or the Ser-Lys-Tyr (SKY) sequence was inserted after the first methionine, synthesized at 23, 30, and 37 °C for 24 h. The synthesized products were analyzed using SDS-PAGE. (**B**) The intensity of the band corresponding to the synthesized ALP was quantified, and the relative amount was calculated as a ratio of the fluorescence of internal EF-Tu. The average of three independent reactions is shown. Error bars indicate standard deviation (S.D).

Next, we investigated whether the Ser-Lys-Tyr sequence in the N-terminal region of sfGFP variants enhances the synthesis of other proteins. We prepared template DNA for the mutants in which Ser-Lys-Tyr (or Ser-Lys-Gly as a control) was inserted after the initiator methionine in DHFR, Luc, and ALP, and synthesized the mutants (Figure 5). The results showed that the synthesis efficiency of ALP significantly increased at all temperatures, especially at 23 °C, when Ser-Lys-Tyr was inserted after Met, whereas the synthesis of Luc and DHFR remained unaffected. This suggests that the N-terminal sequence plays a role in the low productivity of ALP, but not in the low productivity of Luc.

### 2.6. Expression of sfGFP Variants in E. coli

**Figure 6:**
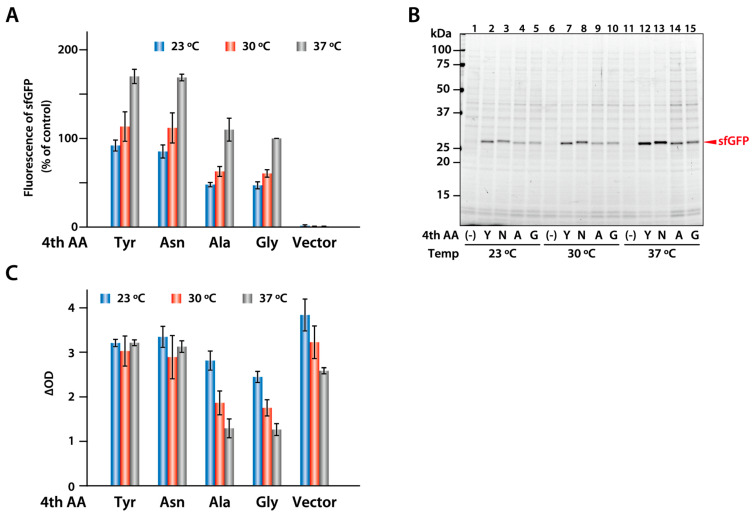
Expression of the four sfGFP variants in *E. coli*. (**A**) sfGFP variants with the indicated amino acid at the fourth position were expressed in *E. coli* growing at 23, 30, and 37 °C, and the fluorescence was measured as described in the Section 4. The relative fluorescence intensity was calculated as a ratio of that of the wild-type sfGFP (glycine at the fourth position) at 37 °C. The average of three independent reactions is shown. Error bars indicate standard deviation (S.D). (**B**) *E. coli* cells expressing the sfGFP variants were analyzed using SDS-PAGE. The sfGFP expressed in *E. coli* grown at 23, 30, and 37 °C is indicated by arrowheads. (**C**) The growth rate of *E. coli* cells expressing sfGFP variants. The growth rate of *E. coli* at OD_600_ (∆OD) was calculated by dividing the OD_600_ at 2 h (30 and 37 °C) or 4 h (23 °C) following the addition of isopropyl β-d-1-thiogalactopyranoside (IPTG) by the OD_600_ prior to IPTG addition. The average of three independent reactions is shown. Error bars indicate standard deviation (S.D).

Finally, we tested whether similar productivity could be obtained when the sfGFP variants are expressed in *E. coli*. The four sfGFP variants were expressed in *E. coli* cultured at 23, 30, and 37 °C using a T7 expression system similar to the PURE system. We found that the tyrosine and asparagine variants, which were synthesized with a high productivity in the PURE system, were also highly expressed in *E. coli*. However, the results differed from those obtained using the PURE system. The yield was highest when the variants were expressed at 37 °C and decreased at lower incubation temperatures (Figure 6A,B). In contrast, the glycine and alanine variants were expressed even at 23 °C, but at lower levels than the tyrosine and asparagine variants at all temperatures (Figure 6A,B). Interestingly, the expression of the glycine and alanine variants suppressed *E. coli* growth at the higher temperatures, whereas the tyrosine and asparagine variants did not (Figure 6C). This suggests that the expression of the glycine and alanine variants induces stress in *E. coli*.

## 3. Discussion

To the best of our knowledge, this is the first study to demonstrate that the amino acid at the fourth position significantly influences translational efficiency in the PURE system, especially at low temperatures. Bivona et al. reported that the amino acid sequence in the N-terminal region affects the expression of recombinant proteins [18]. Ojima-Kato et al. showed that the insertion of a codon encoding the amino acids Ser-Lys-Ile-Lys (SKIK) immediately after the first methionine increased the amount of synthesized scFv [24]. This report is consistent with our result that the productivity of sfGFP with SKI increased compared with that of sfGFP with SKG (Figure 3A). Bivona et al. and Ojima-Kato et al. reported the second amino acid as being important [23,24]; however, in our results, the second amino acid had a smaller effect, and the yield was affected more by the fourth amino acid. The reason for this difference may be that the translational efficiency is affected not only by the N-terminal region but also by the entire ORF sequence. Our results also showed that the addition of SKY to ALP increased its synthesis, whereas its addition to Luc did not (Figure 5). In other words, if the insertion of SKY after the first methionine increases the amount of synthesized protein with low efficiency, the N-terminal sequence is likely responsible for this low efficiency of synthesis.

Most reports on translational efficiency are based on the results of protein synthesis performed at 37 °C, and those that tested synthesis at lower temperatures are limited. However, our results showed a larger difference in the protein amount synthesized at lower temperatures because of the substitution of a single amino acid (Figure 3). Jia et al. reported that large amino acids in the N-terminal region stabilize peptidyl-tRNAs by interacting with ribosomes [27]. Our results showed that larger amino acids promoted translation at low temperatures, suggesting that the interaction of the ribosome with peptidyl-tRNA immediately after initiation is crucial for smooth elongation at low temperatures. At low temperatures, fluctuations in the ribosome are smaller; therefore, smaller amino acids are less likely to place the peptidyl- and aminoacyl-tRNAs in the proper positions to form peptide bonds. Further analysis is required to determine the underlying causes.

When wild-type sfGFP with glycine at the fourth position was expressed in *E. coli*, approximately half of the tyrosine variant was expressed even at 23 °C, in contrast to the synthesis in the PURE system. However, expression at 37 °C was also low, and the growth of *E. coli* was suppressed (Figure 6). Nagao et al. reported that peptidyl-tRNA drop-off occurs frequently during the early stages of translation in *E. coli* cells [28]. In our study, overexpression of wild-type sfGFP at 37 °C resulted in a frequent drop-off of peptidyl-tRNA and a lack of free tRNAs, which affected the growth of *E. coli*. Further studies are needed to elucidate the reason for the difference between synthesis in the PURE system and the expression in *E. coli*; however, expression in *E. coli* may require the translation of proteins other than the target protein, which may have affected *E. coli* growth. On the other hand, this study also suggests that sequences that were synthesized at high levels using the PURE system were efficiently expressed in *E. coli*. Therefore, the PURE system is an effective tool for identifying optimal protein sequences for synthesis, because variants can be easily generated using PCR.

## 4. Materials and Methods

### 4.1. Preparation of Template and Plasmid DNA for Cell-Free Protein Synthesis and Expression in E. coli, Respectively

DHFR and ALP template DNA were amplified from *E. coli* genomic DNA through PCR. For DHFR, the slippery sequence (TTTCCCG) at position 372 was replaced with CTTTCCG. Only the mature region was used for ALP. Wild-type sfGFP and Luc template DNA were designed using the CodHonEditor [25] based on *E. coli* codon usage and synthesized by Eurofins Genomics (Tokyo, Japan). Trastuzumab HC DNA was designed and synthesized by GenScript (Tokyo, Japan). The 5′-UTR (5′-GAAATTAATACGACTCACTATAGGGAGACCACAACGGTTTCCCTCTAGAAATAATTTTGTTTAACTTTAAGAAGGAGATATACCA-ORF-3′), containing the T7 promoter and the Shine–Dalgarno (SD) sequence, and the 3′-UTR (5′-ORF-TAATGAATAACTAATCC-3′) were added to all template DNA, and the template DNA was synthesized through PCR. The sequences of the template DNA for DHFR, ALP, sfGFP, Luc, and trastuzumab HC are shown in Tables S1–S3. Template DNA for trastuzumab HC and sfGFP variants containing different N-terminal sequences was prepared through PCR using primers containing the corresponding nucleotide sequence. For expression in *E. coli*, the sfGFP variant DNA was inserted into the NcoI and XhoI sites in the pET-21d vector using the In-Fusion^®^ Cloning Kit (TakaraBio, Shiga, Japan), and a Gly-Gly-Gly-Gly-Ser linker was inserted between the sfGFP and hexahistidine tags using inverse PCR.

### 4.2. Protein Synthesis Using the PURE System

The PURE*frex*^®^ *2.1* Kit (GeneFrontier, Chiba, Japan) was used as the PURE system reagent for cell-free protein synthesis. For protein synthesis, 5 or 10 µL of the reaction mixture containing 1 (DHFR, sfGFP, and trastuzumab HC) or 2 (Luc and ALP) ng/µL of template DNA was incubated at 23, 30, and 37 °C for the indicated duration, as described in the corresponding figure legends. Following synthesis, the reaction mixture was diluted 50- or 100-fold with water, and the fluorescence of the synthesized sfGFP was measured using a Varioskan™ plate reader (Thermo Scientific, Waltham, MA, USA). For SDS-PAGE analysis, 0.2 or 0.5 µL of the reaction mixture was loaded onto 12.5% or 10–20% gradient gel, and electrophoresis was performed under reducing condition. The gels were stained with Oriole (Bio-Rad, Hercules, CA, USA) or SYPRO Orange Protein Gel Stain (Thermo Scientific). Protein bands were visualized and quantified using ImageQuant Las imager (Cytiva, Tokyo, Japan) or WSE-6300 LuminoGraph III (ATTO, Tokyo, Japan) and CS Analyzer 4 software (ATTO).

### 4.3. Expression of sfGFP in E. coli

*E. coli* BL21 (DE3) cells were transformed with a plasmid containing sfGFP variant DNA. The transformants were incubated in Terrific Broth (TB) medium supplemented with 50 µg/mL of carbenicillin at 37 °C overnight. The overnight culture was used to inoculate fresh TB medium with OD_600_ of 0.1 and incubated at 23, 30, and 37 °C. When OD_600_ reached 1, 0.1 mM of IPTG was added and further incubated for 2 h at 30 and 37 °C or for 4 h at 23 °C. To measure fluorescence, 0.005 OD_600_ units in 100 µL of medium were analyzed using a Varioskan™ plate reader (Thermo Scientific). For SDS-PAGE analysis, 0.0005 OD_600_ units were subjected to 10–20% gradient gel electrophoresis. After electrophoresis, gels were stained with SYPRO Orange Protein Gel Stain (Thermo Scientific). Protein bands were visualized and quantified using a WSE-6300 LuminoGraph III (ATTO) and CS Analyzer 4 software (ATTO).

## 5. Conclusions

This study demonstrated that even a single amino acid substitution in the N-terminal region significantly affects translational efficiency, especially at a low temperature, which will be an important validation in the expression of the protein of interest both in vitro and in vivo.

## Data Availability

Data are contained within the article.

## Supplementary Materials

The following supporting information can be downloaded at: https://www.mdpi.com/article/10.3390/ijms25105264/s1.
