# Supplementary material for: N-Terminal Amino Acid Affects the Translation Efficiency at Lower Temperatures in a Reconstituted Protein Synthesis System
Source: Int J Mol Sci. 2024 May 12;25(10):5264. doi: 10.3390/ijms25105264 (PMC11120837; doi:10.3390/ijms25105264)
Supplement: Supplementary file 1 [file ijms-25-05264-s001.zip › ijms-2985240-supplementary.pdf]

Table S1. Sequence of DHFR and ALP derived from *E. coli* genome

Name	Amino acid sequence[*1]	Nucleotide sequence[*2, 3]
DHFR	MISLIAALAVDRVIGMENAMPWNLPADLAWFKRNTLNKPVIMGRHTW<br>ESIGRPLPGRKNIILSSQPGTDDRVTWVKSVDEAIAACGDVPEIMVI<br>GGGRVYEQFLPKAQKLYLTHIDAEVEGDTHFPDYEPDDWESVFSEFH<br>DADAQNSHSYCFEILERR	gaaattaatacgactcactatagggagaccacaacggtttccctctagaaataattttgtttaactttaagaaggagatatacca<br>ATGATCAGTCTGATTGCGGCGTTAGCGGTAGATCGCGTTATCGGCATGGAAAACGCCATGCCGTGGAACCTGCCTGCCGATCTCG<br>CCTGGTTTAAACGCAACACCTTAAATAAACCCGTGATTATGGGCCGCCATACCTGGGAATCAATCGGTCGTCCGTTGCCAGGACG<br>CAAAAATATTATCCTCAGCAGTCAACCGGGTACGGACGATCGCGTAACGTGGGTGAAGTCGGTGGATGAAGCCATCGCGGCGTGT<br>GGTGACGTACCAGAAATCATGGTGATTGGCGGCGGTCGCGTTTATGAACAGTTCTTGCCAAAAGCGCAAAAACTGTATCTGACGC<br>ATATCGACGCAGAAGTGGAAGGCGACACCCACTTTCCGGATTACGAGCCGGATGACTGGGAATCGGTATTCAGCGAATTCCACGA<br>TGCTGATGCGCAGAACTCTCACAGCTATTGCTTTGAGATTCTGGAGCGGCGGtaatgaataactaatcc
ALP	MRTPEMPVLENRAAQGDITAPGGARRLTGDQTAALRDSLSDKPAKNI<br>ILLIGDGMGDSEITAARNYAEGAGGFFKGIDALPLTGQYTHYALNKK<br>TGKPDYVTDSAASATAWSTGVKTYNGALGVDIHEKDHPTILEMAKAA<br>GLATGNVSTAELQDATPAALVAHVTSRKCYGPSATSEKCPGNALEKG<br>GKGSITEQLLNARADVTLGGGAKTFAETATAGEWQGKTLREQAQARG<br>YQLVSDAASLNSVTEANQQKPLLGLFADGNMPVRWLGPKATYHGNID<br>KPAVTCTPNPQRNDSVPTLAQMTDKAIELLSKNEKGFFLQVEGASID<br>KQDHAANPCGQIGETVDLDEAVQRALEFAKKEGNTLVIVTADHAHAS<br>QIVAPDTKAPGLTQALNTKDGAVMVMSYGNSEEDSQEHTGSQLRIAA<br>YGPHAANVVGLTDQTDLFYTMKAALGLK	gaaattaatacgactcactatagggagaccacaacggtttccctctagaaataattttgtttaactttaagaaggagatatacca<br>ATGCGGACACCAGAAATGCCTGTTCTGGAAAACCGGGCTGCTCAGGGCGATATTACTGCACCCGGCGGTGCTCGCCGTTTAACGG<br>GTGATCAGACTGCCGCTCTGCGTGATTCTCTTAGCGATAAACCTGCAAAAAATATTATTTTGCTGATTGGCGATGGGATGGGGGA<br>CTCGGAAATTACTGCCGCACGTAATTATGCCGAAGGTGCGGGCGGCTTTTTTAAAGGTATAGATGCCTTACCGCTTACCGGGCAA<br>TACACTCACTATGCGCTGAATAAAAAAACCGGCAAACCGGACTACGTCACCGACTCGGCTGCATCAGCAACCGCCTGGTCAACCG<br>GTGTCAAAACCTATAACGGCGCGCTGGGCGTCGATATTCACGAAAAAGATCACCCAACGATTCTGGAAATGGCAAAAGCCGCAGG<br>TCTGGCGACCGGTAACGTTTCTACCGCAGAGTTGCAGGATGCCACGCCCGCTGCGCTGGTGGCACATGTGACCTCGCGCAAATGC<br>TACGGTCCGAGCGCGACCAGTGAAAAATGTCCGGGTAACGCTCTGGAAAAAGGCGGAAAAGGATCGATTACCGAACAGCTGCTTA<br>ACGCTCGTGCCGACGTTACGCTTGGCGGCGGCGCAAAAACCTTTGCTGAAACGGCAACCGCTGGTGAATGGCAGGGAAAAACGCT<br>GCGTGAACAGGCACAGGCGCGTGGTTATCAGTTGGTGAGCGATGCTGCCTCACTGAATTCGGTGACGGAAGCGAATCAGCAAAAA<br>CCCCTGCTTGGCCTGTTTGCTGACGGCAATATGCCAGTGCGCTGGCTAGGACCGAAAGCAACGTACCATGGCAATATCGATAAGC<br>CCGCAGTCACCTGTACGCCAAATCCGCAACGTAATGACAGTGTACCAACCCTGGCGCAGATGACCGACAAAGCCATTGAATTGTT<br>GAGTAAAAATGAGAAAGGCTTTTTCCTGCAAGTTGAAGGTGCGTCAATCGATAAACAGGATCATGCTGCGAATCCTTGTGGGCAA<br>ATTGGCGAGACGGTCGATCTCGATGAAGCCGTACAACGGGCGCTGGAATTCGCTAAAAAGGAGGGTAACACGCTGGTCATAGTCA<br>CCGCTGATCACGCCCACGCCAGCCAGATTGTTGCGCCGGATACCAAAGCTCCGGGCCTCACCCAGGCGCTAAATACCAAAGATGG<br>CGCAGTGATGGTGATGAGTTACGGGAACTCCGAAGAGGATTCACAAGAACATACCGGCAGTCAGTTGCGTATTGCGGCGTATGGC<br>CCGCATGCCGCCAATGTTGTTGGACTGACCGACCAGACCGATCTCTTCTACACCATGAAAGCCGCTCTGGGGCTGAAAtaatgaa<br>taactaatcc

1. For ALP, only the mature region was used.
2. ORFs are shown by uppercase. T7 promoter and SD sequence are underlined and double-lined, respectively.
3 For DHFR, a slippery sequence (TTTCCCG) at position 372 was substituted with CTTTCCG indicated by red letters..

Table S2. Sequence of sfGFP and Luc

Name	Amino acid sequence	Nucleotide sequence[*1]
sfGFP	MSKGEELFTGVVPILVELDGDVNGHKFSVRGEGEGDATNGKLTLKFI<br>CTTGKLPVPWPTLVTTLTYGVQCFSRYPDHMKRHDFFKSAMPEGYVQ<br>ERTISFKDDGTYKTRAEVKFEGDTLVNRIELKGIDFKEDGNILGHKL<br>EYNFNSHNVYITADKQKNGIKANFKIRHNVEDGSVQLADHYQQNTPI<br>GDGPVLLPDNHYLSTQSVLSKDPNEKRDHMVLLEFVTAAGITHGMDE<br>LYK	gaaattaatacgactcactatagggagaccacaacggtttccctctagaaataattttgtttaactttaagaaggagatatacca<br>ATGTCTAAAGGTGAAGAATTATTTACTGGTGTTGTGCCGATCCTGGTCGAACTGGACGGTGATGTGAATGGGCATAAATTCTCGG<br>TTCGGGGCGAAGGAGAGGGTGACGCAACTAACGGCAAACTTACCCTCAAGTTTATTTGTACCACAGGTAAACTGCCAGTCCCGTG<br>GCCCACGCTGGTGACCACTTTGACCTACGGCGTACAGTGCTTCAGCCGCTATCCGGATCACATGAAACGTCATGATttcTTCAAA<br>TCAGCGATGCCTGAAGGGTATGTTCAGGAACGCACGATTAGCTTTAAAGACGATGGCACCTACAAGACACGTGCCGAGGTGAAAT<br>TTGAAGGTGATACGTTAGTCAATCGCATCGAACTGAAAGGCATTGACTTCAAAGAGGATGGAAACATCCTGGGTCATAAGCTGGA<br>ATATAACTTTAATTCTCACAACGTGTACATTACCGCTGACAAACAAaagAATGGCATCAAAGCGAACTTCAAGATTCGTCATAAT<br>GTTGAAGATGGGTCCGTACAGCTTGCAGATCACTATCAGCAAAACACTCCGATCGGTGACGGCCCAGTGCTCCTGCCGGATAATC<br>ATTACTTGAGTACCCAGTCGGTCTTAAGCAAAGATCCGAACGAGAAACGCGACCACATGGTTCTGCTGGAATTTGTGACGGCCGC<br>GGGTATTACCCATGGCATGGATGAACTGTATAAAtaatgaataactaatcc
Luc	MEDAKNIKKGPAPFYPLEDGTAGEQLHKAMKRYALVPGTIAFTDAHI<br>EVNITYAEYFEMSVRLAEAMKRYGLNTNHRIVVCSENSLQFFMPVLG<br>ALFIGVAVAPANDIYNERELLNSMNISQPTVVFVSKKGLQKILNVQK<br>KLPIIQKIIIMDSKTDYQGFQSMYTFVTSHLPPGFNEYDFVPESFDR<br>DKTIALIMNSSGSTGLPKGVALPHRTACVRFSHARDPIFGNQIIPDT<br>AILSVVPFHHGFGMFTTLGYLICGFRVVLMYRFEEELFLRSLQDYKI<br>QSALLVPTLFSFFAKSTLIDKYDLSNLHEIASGGAPLSKEVGEAVAK<br>RFHLPGIRQGYGLTETTSAILITPEGDDKPGAVGKVVPFFEAKVVDL<br>DTGKTLGVNQRGELCVRGPMIMSGYVNNPEATNALIDKDGWLHSGDI<br>AYWDEDEHFFIVDRLKSLIKYKGYQVAPAELESILLQHPNIFDAGVA<br>GLPDDDAGELPAAVVVLEHGKTMTEKEIVDYVASQVTTAKKLRGGVV<br>FVDEVPKGLTGKLDARKIREILIKAKKGGKSKL	gaaattaatacgactcactatagggagaccacaacggtttccctctagaaataattttgtttaactttaagaaggagatatacca<br>ATGGAAGATGCTAAAAATATTAAAAAAGGTCCAGCGCCCTTTTATCCTCTGGAGGACGGTACTGCTGGGGAACAGCTTCACAAAG<br>CAATGAAACGTTACGCCCTGGTGCCGGGCACCATCGCGTTCACTGATGCTCACATTGAAGTCAACATCACGTACGCAGAATATTT<br>CGAGATGTCTGTACGCCTGGCTGAAGCGATGAAAAGGTACGGTCTCAACACCAACCATCGAATTGTTGTGTGTAGCGAAAATTCC<br>CTGCAGTTTTTCATGCCGGTTCTGGGCGCCCTGTTTATCGGTGTAGCTGTTGCACCGGCGAACGACATCTACAACGAACGTGAGT<br>TACTTAACAGTATGAATATTTCACAGCCAACAGTCGTGTTCGTTTCTAAGAAAGGACTGCAAAAAATCCTGAACGTACAGAAAAA<br>GCTGCCGATCATTCAGAAAATCATCATAATGGATAGCAAAACTGACTATCAAGGCTTCCAGTCCATGTACACCTTTGTTACTTCG<br>CACTTGCCTCCGGGCTTCAACGAATATGATTTCGTGCCGGAATCTTTTGACCGTGATAAAACGATTGCTCTCATCATGAACAGCT<br>CTGGTTCCACCGGCCTGCCCAAGGGGGTAGCACTGCCACATCGCACTGCTTGCGTTCGTTTCAGTCACGCCCGCGACCCGATTTT<br>CGGTAATCAGATCATCCCTGATACCGCGATTCTGAGCGTCGTTCCGTTTCACCATGGCTTCGGTATGTTCACTACGCTTGGCTAC<br>CTGATCTGTGGTTTTCGTGTAGTGTTAATGTACCGGTTCGAGGAAGAACTGTTTCTGAGATCTCTGCAGGACTATAAAATCCAAT<br>CCGCTCTCCTGGTTCCGACCTTGTTCTCATTCTTTGCAAAATCTACTCTGATTGATAAATACGACCTTAGCAACCTGCACGAAAT<br>CGCTTCGGGCGGTGCGCCGCTGTCTAAGGAGGTAGGGGAAGCAGTTGCCAAACGTTTCCATTTACCAGGAATCCGCCAGGGTTAC<br>GGCCTGACCGAAACAACTTCCGCTATTCTGATCACGCCTGAGGGTGACGATAAACCGGGCGCGGTGGGTAAGGTCGTTCCCTTCT<br>TTGAAGCTAAAGTAGTGGACCTCGATACCGGCAAAACTCTGGGTGTTAACCAGCGTGGGGAACTTTGCGTACGCGGCCCGATGAT<br>TATGAGTGGTTATGTTAACAATCCGGAAGCAACCAACGCTCTGATCGACAAAGATGGCTGGCTGCACAGCGGTGACATCGCCTAC<br>TGGGATGAGGACGAACACTTCTTCATTGTCGATCGTCTGAAGTCTTTGATCAAATACAAAGGATATCAAGTGGCGCCAGCAGAAC<br>TGGAGTCCATCTTACTGCAGCATCCTAACATTTTTGACGCTGGTGTTGCGGGCCTCCCGGATGACGATGCTGGTGAACTGCCGGC<br>AGCCGTAGTTGTGCTTGAACACGGGAAAACTATGACGGAAAAGGAGATCGTAGACTACGTTGCTAGCCAGGTCACCACTGCGAAA<br>AAACTGCGTGGCGGTGTTGTGTTCGTAGATGAAGTTCCGAAAGGCCTGACCGGTAAGCTGGACGCACGCAAAATCCGTGAAATTC<br>TGATCAAAGCTAAAAAAGGCGGTAAATCTAAACTGtaatgaataactaatcc

1. ORFs are shown by uppercase. T7 promoter and SD sequence are underlined and double-lined, respectively.

Table S3. Sequence of trastuzumab HC (AT variant)

Name	Amino acid sequence	Nucleotide sequence*1
AT	MEVQLVESGGGLVQPGGSLRLSCAASGFNIKDTYIHWVRQAPGKGLE<br>WVARIYPTNGYTRYADSVKGRFTISADTSKNTAYLQMNSLRAEDTAV<br>YYCSRWGGDGFYAMDYWGQGTLVTVSSASTKGPSVFPLAPSSKSTSG<br>GTAALGCLVKDYFPEPVTVSWNSGALTSGVHTFPAVLQSSGLYSLSS<br>VVTVPSSSLGTQTYICNVNHKPSNTKVDKKVEPKS	gaaattaatacgactcactatagggagaccacaacggtttccctctagaaataattttgtttaactttaagaaggagatatacca<br>ATGGAAGTACAATTAGTTGAATCGGGTGGTGGTCTGGTCCAACCGGGTGGCTCGCTGCGTCTGTCGTGTGCGGCGTCGGGCTTTA<br>ACATCAAAGATACCTATATTCATTGGGTTCGTCAGGCACCGGGCAAAGGTCTGGAATGGGTCGCTCGCATCTACCCGACCAACGG<br>CTATACGCGTTACGCGGATTCTGTGAAAGGTCGCTTTACCATTTCCGCGGACACCTCAAAAAACACGGCCTATCTGCAGATGAAC<br>AGCCTGCGTGCAGAAGACACGGCTGTTTATTACTGCAGTCGCTGGGGCGGTGATGGCTTTTATGCCATGGACTACTGGGGCCAAG<br>GTACCCTGGTCACCGTGAGCTCTGCATCGACCAAAGGTCCGAGCGTCTTCCCGCTGGCTCCGAGTTCCAAATCGACCAGCGGCGG<br>TACGGCAGCACTGGGTTGTCTGGTTAAAGATTATTTTCCGGAACCGGTTACCGTCTCTTGGAACAGTGGCGCGCTGACCAGCGGT<br>GTCCATACGTTCCCGGCAGTGCTGCAGTCATCGGGTCTGTATAGCCTGAGCTCTGTGGTTACCGTGCCGAGTTCCTCACTGGGTA<br>CCCAAACGTACATCTGCAACGTTAATCACAAACCGTCCAATACGAAAGTGGATAAAAAAGTTGAACCGAAAAGTtaatgaataac<br>taatcc

1. ORF is shown by uppercase. T7 promoter and SD sequence are underlined and double-lined, respectively.
